# *INF2* p.Arg214Cys mutation in a Chinese family with rapidly progressive renal failure and follow-up of renal transplantation: case report and literature review

**DOI:** 10.1186/s12882-021-02254-9

**Published:** 2021-02-04

**Authors:** Wenbo Zhao, Xinxin Ma, Xiaohao Zhang, Dan Luo, Jun Zhang, Ming Li, Zengchun Ye, Hui Peng

**Affiliations:** 1 Division of Nephrology, The Third Affiliated Hospital of Sun Yat-sen University, Tianhe Ave #600, Guangzhou, 510630 China; 2 Department of Nephrology, The First Affiliated Hospital of Sun Yat-sen University, Guangzhou, Guangdong China

**Keywords:** *INF2*, Mutation analysis, End-stage renal disease, Kidney transplant

## Abstract

**Background:**

Heterozygous mutations in the inverted formin 2 (*INF2*) gene are related to secondary focal segmental glomerulosclerosis (FSGS), a rare secondary disease associated with rapidly progressive renal failure.

**Case presentation:**

We report a patient with familial autosomal *INF2* mutation manifesting nephritic syndromes and elevated serum creatinine levels. Mutational analysis revealed an autosomal dominant (AD) inheritance pattern and a mutation in exon 4 (p.Arg214Cys) of *INF2* as the likely cause, which has not been previously described in an Asian family. The patient progressed to end-stage renal disease (ESRD) and received hemodialysis. His mother had undergone renal transplant 3 years earlier, and his grandmother had carried the p.Arg214Cys mutation for more than 80 years without any sign of renal dysfunction.

**Conclusions:**

This is the first report to identify an association between a familial autosomal dominant *INF2* p.Arg214Cys mutation and rapidly progressive renal disease in an Asian family. INF2 mutation analysis should not be restricted to individuals without family history of FSGS, rather it should also be performed on individuals for whom drug-based therapies are not effective. In this case, kidney transplant is an effective alternative.

## Background

Formins are widely expressed proteins that govern remodeling of the actin cytoskeleton during cytokinesis, cell polarization, and tissue morphogenesis [1].

In the last 10 years, the inverted formin 2 (*INF2)* has been an important target of mutations responsible for focal segmental glomerulosclerosis (FSGS) [2]. Brown et al. [3] identified an association between heterozygous mutations in *INF2* gene and FSGS in 12% of the families studied and recorded the ages of affected individuals at the time of diagnosis and the time of developing ESRD. *INF2* encodes an actin regulatory protein of the formin family, which plays an important role in maintaining podocyte plasticity [4, 5].

*INF2* mutations have been identified as being responsible for the development of autosomal dominant (AD) FSGS [3, 4, 6, 7]. These mutations are also associated with Charcot-Marie-Tooth disease (CMT) [2, 5, 8–11], which is characterized by a demyelinating peripheral neuropathy [9]. In CMT patients, an increased prevalence of FSGS has been documented [12]. While the incidence of FSGS is one in a million of the general population, its incidence is one out of four hundred in CMT patients [13]. *INF2* mutations are thought to be the link between FSGS and CMT: the frequency of *INF2* mutation in patients with both FSGS and CMT is much higher (75%) than that in patients affected by FSGS alone (12–17%) [3, 14, 15]; no *INF2* mutations were found in patients with CMT alone. However, it has not been clearly explained why *INF2* mutation does not always lead to nerve phenotypic abnormalities. Boyer et al. identified INF2 mutations in exons 2 and 3 in 75% of patients with both CMT and FSGS [5]. De Jonghe et al. reported that mutations in exon 3 of *INF2* are responsible for causing CMT-FSGS, whereas mutations in exon 4 and 6 are associated with FSGS alone [10]. However, Caridi et al. reported that CMT patients carried a mutation in exon 4 [9]. Quantitative live-cell imaging may identify distinct subsets of *INF2* variants that are linked to FSGS alone or to CMT-FSGS.

In terms of clinical outcomes, intra-familial phenotypes of *INF2* mutation are varied. Lee et al. [16] and Caridi et al. [9] observed marked clinical heterogeneity and different severities of disease within families carrying the same *INF2* mutation. Due to incomplete penetrance, family members that carry autosomal dominant FSGS may also be asymptomatic [16]. Although more than 70 families and 250 patients have been reported to carry an *INF2* mutation in previous reports [4, 9, 17], the affected families are all of European descent. Here, we report a new familial autosomal *INF2* mutation in exon 4 (p.Arg214Cys) present in two patients from the same three-generation family of Chinese origin; these patients presented with proteinuria, high blood pressure, and hyperuricemia, and exhibited rapid progression of renal failure. This is the first report of *INF2* p.Arg214Cys in Asian families, and one of the few reported cases worldwide.

## Case presentation

We report the case of a 23-year-old male patient admitted to our hospital in June 2018. The patient had a 4-month history of foamy urine prior to admission. His medical report revealed proteinuria (+++), mild edema of the lower limbs, elevated serum creatinine (259 μmol/L) levels, and mild hypertension (145/78 mmHg). The patient had no prior history of renal problems.

The patient was hospitalized due to nephrotic syndrome and renal failure. Laboratory testing revealed the following: blood urea nitrogen (BUN), 7.1 mmol/L; serum creatinine (Scr), 282 μmol/L; urine albumin/creatinine (UACR), 2701.53 mg/g; urine protein/creatinine (UPCR), 3360 mg/g; urinary protein quantitative (24 h), 6.678 g; serum albumin, 34.3 g/L; serum uric acid, 561 μmol/L; total cholesterol, 7.63 mmol/L; low density lipoprotein, 5.18 mmol/L; hemoglobin (Hb), 130 g/L; anti-PLA2R, negative; parathyroid hormone (iPTH), 135.79 pg/mL; and homocysteine (HCY), 22.23 μmol/L. Interventricular septal thickness was found to be 12 mm using cardiac ultrasound. As renal ultrasound revealed renal cortex thinning and an unclear boundary of the renal cortex and medulla, renal biopsy was not performed.

After discharge, the patient was prescribed *α-keto acid,* atorvastatin calcium, and febuxostat tablets to control proteinuria and edema. Unfortunately, the patient’s proteinuria was not improved, and his serum creatinine levels rose rapidly (Table 1). Prednisone (Starting dose 1 mg/kg) was administered for several months but was not effective as renal failure rapidly progressed. In March 2020, the patient started hemodialysis. In June 2020, the patient underwent renal transplantation. The transplantation was successful, and the patient has not shown any sign of proteinuria or hematuria since.

**Table 1:** Time course of levels of laboratory testing of the patient

	2018				2019					2020	
	Feb	Jun	Aug	Sep	Mar	Apr	May	Sep	Nov	Jan	Mar
Serum creatinine (μmol/L)	259	298	240	245	300	329	339	457	526	582	2000
Urine albumin/creatinine (mg/g)		5009.26	2522.07	2356.13	2771.14	2528.06	2587.36	2587.36		3620	
Urine protein/creatinine (mg/g)		5487	3046	2844	2905	3012	2641	2641		3656	
Serum uric acid (μmol/L)	467	583	159	222	351	390	400	346	367	294	
Hemoglobin (g/L)		130				114			108	92	78

Eight years ago, the patient’s mother had manifested proteinuria, elevated serum creatinine levels (142 μmol/L), high blood pressure, and hyperuricemia. Angiotensin receptor blockers (ARBs) treatment was ineffective, and renal failure rapidly progressed. Peritoneal dialysis was initiated 3 years later (12/31/2014), and kidney transplantation was performed after another 2 years (03/03/2017). After transplantation, proteinuria was completely resolved; blood pressure and uric acid returned to normal; renal function improvement was also recorded (recent serum creatinine, 114 μmol/L) (Table 2).

**Table 2:** Laboratory test of the patient’s mother before initiating hemodialysis

	2012	2013	2014				2015		2016	2017
	Oct	Dec	Feb	Apr	Jul	Dec	Feb	May	Jul	Feb
Serum creatinine (μmol/L)	214	342	477	463.26	559.57	643	454	570.7	1263	1443
Urine protein	3+	3+	3+	3+	2+	2+	3+	3+	3+	3+
Serum uric acid (μmol/L)	/	/	704	755	631	678	464	437	438.8	489
Hemoglobin (g/L)	130	/	102	98	96	60	81	106	111	62

Qualitative analysis of albuminuria: -, 1+, 2+, 3+, and 4+

**Fig. 1:**
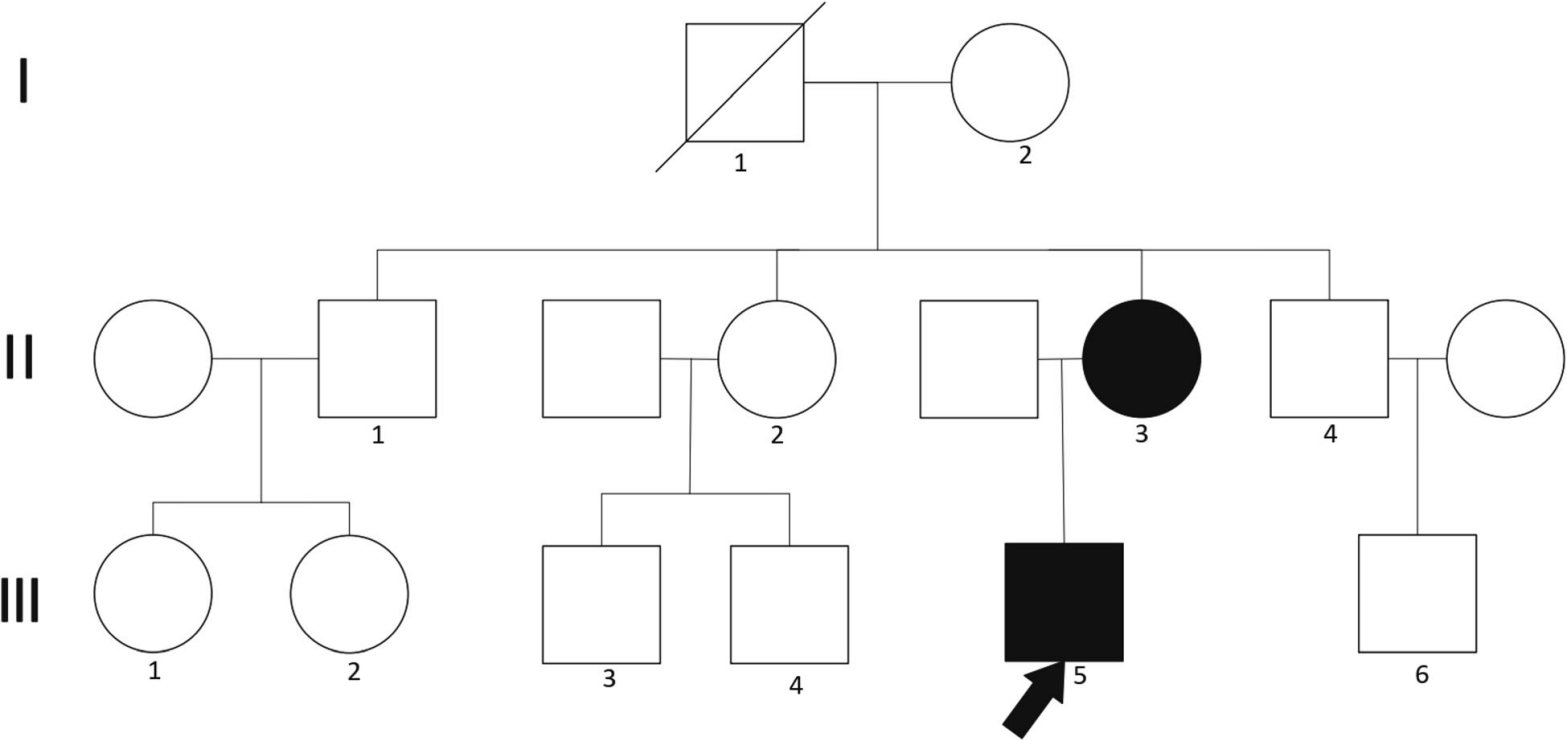
Pedigree of a family with the INF2 p.Arg214Cys mutation (I2, II3, III5). The arrow indicates the problem (III5). Black boxes (II3, III5) indicate the affected members of the family related kidney disease. White boxes indicates a genetically tested patient with a normal result

**Fig. 2:**
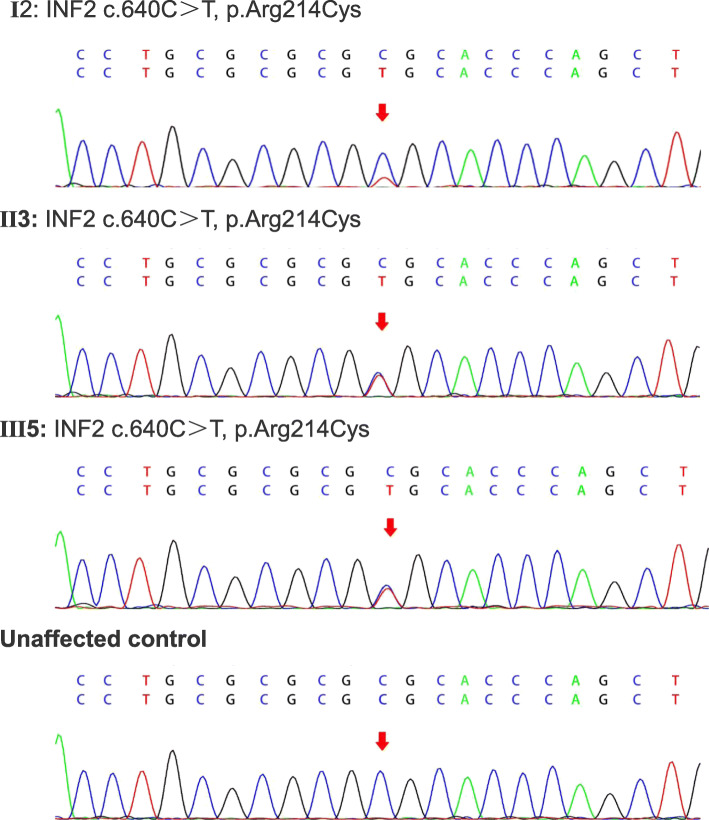
Sequence alterations in members (I2, II3, III5) of families. Sequencing in families INF2 gene was obtained from amplified genomic DNA

Given the patient’s familial history of renal failure, whole exome sequencing (WES) was performed. Genomic DNA from the patient, his mother, and a control was extracted from buccal swab samples, using a QIAmp DNA Mini Kit (Qiagen China Co., Ltd., Shanghai, China). Target capture sequencing was performed on the extracted DNA. All coding exons were enriched. Genetic analysis, variation annotation, and identification of candidate pathogenic mutations were performed. For our study, a total of 16 members of the patient’s family were recruited. Three individuals were affected in this pedigree, including the patient, his mother, and his maternal grandmother. Sequencing of exon 4 of autosomal *INF2* revealed a nonsynonymous missense variant, wherein a C was substituted with T (c.640C > T), resulting in the p.Arg214Cys mutation (Figs. 1 and 2).

We conducted a review of the literature to compare the phenotypes of our patients with other patients reported to have been carrying the *INF2* p.Arg214Cys mutation (Table 3).

**Table 3:** Comparison of phenotypes between the present patients with INF2 p.Arg214Cys and previously reported patients

Family	Countries/region	Familial/Sporadic	cDNA Mut	Prot Mut	Exon	Number of cases with proteinuria	Proteinuria (g/L) median	Histology	CMT	Number of healthy cases	Age at disease onset	Number of cases of ESRD
1	Europe [4]	Familial	c.640C > T	p.Arg214Cys	4	5	/	FSGS	0	1	5–44	3
2	Europe [4]	Familial	c.640C > T	p.Arg214Cys	4	2	/	FSGS	0	0	16–37	2
3	Australia [17]	Familial	c.640C > T	p.Arg214Cys	4	2	/	FSGS	0	0	21-?	1
4	Italian [9]	Familial	c.640C > T	p.Arg214Cys	4	3	1.6	FSGS	0	1	15–26	1
5	China	Familial	c.640C > T	p.Arg214Cys	4	2	>3.5		0	1	23–40	2

*CMT* Charcot-Marie-Tooth disease, *ESRD* End-stage renal disease

## Discussion and conclusions

We report a familial autosomal mutation in exon 4 of *INF2* (p.Arg214Cys) in two patients from the same three-generation family of Chinese origin. Informed consent was provided by the patient. Two patients (son and mother) manifested proteinuria, high blood pressure, and hyperuricemia, and exhibited rapid progression of renal failure, which may be related to FSGS, and ultimately progressed to ESRD. In both cases, kidney transplantation was effective, while drug-based therapies were not.

Several studies have reported a familial autosomal mutation in exon 4 of *INF2* (p.Arg214Cys) in patients of Caucasian descent (Table 3). The patients reported were relatively young at the time of diagnosis and at ESRD. Some carriers progressed to ESRD, while others were healthy. In our study, kidney dysfunction progressed rapidly in the patient and the patient’s mother. The patient’s grandmother carried an *INF2* mutation without any sign of renal dysfunction. In our two clinical cases—son and mother—the age of symptoms onset was 20 and 40 years old, respectively, and no symptoms of CMT were observed. This may be due to the autosomal mutation located in exon 4 of *INF2* [9, 10]. Current literature describing patients with p.Arg214Cys mutations reports the same phenotype but without CMT symptoms. In a study on a family of a father and two sons, Lee et al. reported diseases of very different severity with the same *INF2* mutation [16]. In our cases, we also observed that the patient’s grandmother, who carried an *INF2* mutation without any sign of renal dysfunction, is consistent with reports of patients of Caucasian descent. However, it is worth noting that previously reported healthy carriers were younger, thus a delayed onset is possible. Therefore, our case confirms the existence of healthy carriers. Some families showed marked clinical heterogeneity, suggesting the involvement of environmental factors or a specific genetic background [5].

In our study, the son and the mother both presented complications such as elevated blood pressure and serum uric acid levels, which may be the prominent phenotype. In general, corticosteroids are used to treat idiopathic FSGS. However, corticosteroid therapy is not effective for genetic FSGS [2, 13]. In our case, before verifying INF2 mutation with WES, corticosteroids in combination with angiotensin receptor blockers (ARBs) were used for treatment, which were ineffective for managing genetic disorders [13].

The patient’s mother had undergone renal transplantation 3 years ago. In June 2020, the patient underwent renal transplantation. After renal transplantation, the patient’s proteinuria was completely resolved; his blood pressure and uric acid returned to normal; his renal function improved as the transplanted kidney functioned properly. This indicates that renal transplantation is an effective treatment for this condition [2].

To our knowledge, this is the first report on *INF2* p.Arg214Cys in Asia and one of the few reports worldwide. In addition to confirming previously reported characteristics associated with *INF2* mutation, our study also suggests new clinical manifestations and treatment outcomes that are worthy of note. INF2 mutation analysis should not be restricted to individuals without family history of FSGS, rather it should also be performed on individuals for whom drug-based therapies are not effective. For these patients, Kidney transplantation is an effective treatment.

## Data Availability

The datasets used and analyzed during the course of the current study are available from the corresponding author on reasonable request.

## Abbreviations

*INF2*: Inverted formin 2 gene
FSGS: Focal segmental glomerulosclerosis
ESRD: End-stage renal disease
AD: Autosomal dominant
CMT: Charcot-Marie-Tooth disease
UACR: Urine albumin/creatinine
ARB: Angiotensin receptor blockers
HCY: Homocysteine
WES: Whole exome sequencing

## References

[1] Goode BL, Eck MJ (2007). Mechanism and function of formins in the control of actin assembly. Annu Rev Biochem.

[2] Labat-de-Hoz L, Alonso MA (2020). The formin INF2 in disease: progress from 10 years of research. Cell Mol Life Sci.

[3] Brown EJ, Schlöndorff JS, Becker DJ, Tsukaguchi H, Tonna SJ, Uscinski AL (2010). Mutations in the formin gene INF2 cause focal segmental glomerulosclerosis. Nat Genet.

[4] Boyer O, Benoit G, Gribouval O, Nevo F, Tête MJ, Dantal J (2011). Mutations in INF2 are a major cause of autosomal dominant focal segmental glomerulosclerosis. J Am Soc Nephrol.

[5] Boyer O, Nevo F, Plaisier E, Funalot B, Gribouval O, Benoit G (2011). INF2 mutations in Charcot-Marie-tooth disease with glomerulopathy. N Engl J Med.

[6] Gbadegesin RA, Lavin PJ, Hall G, Bartkowiak B, Homstad A, Jiang R (2012). Inverted formin 2 mutations with variable expression in patients with sporadic and hereditary focal and segmental glomerulosclerosis. Kidney Int.

[7] Münch J, Grohmann M, Lindner TH, Bergmann C, Halbritter J (2016). Diagnosing FSGS without kidney biopsy - a novel INF2-mutation in a family with ESRD of unknown origin. BMC Med Genet.

[8] Echaniz-Laguna A, Latour P (2019). A cryptic splicing mutation in the INF2 gene causing Charcot-Marie-tooth disease with minimal glomerular dysfunction. J Peripher Nerv Syst.

[9] Caridi G, Lugani F, Dagnino M, Gigante M, Iolascon A, Falco M (2014). Novel INF2 mutations in an Italian cohort of patients with focal segmental glomerulosclerosis, renal failure and Charcot-Marie-tooth neuropathy. Nephrol Dial Transplant.

[10] Mademan I, Deconinck T, Dinopoulos A, Voit T, Schara U, Devriendt K (2013). De novo INF2 mutations expand the genetic spectrum of hereditary neuropathy with glomerulopathy. Neurology.

[11] Bayraktar S, Nehrig J, Menis E, Karli K, Janning A, Struk T (2020). A deregulated stress response underlies distinct INF2-associated disease profiles. J Am Soc Nephrol.

[12] Paul MD, Fernandez D, Pryse-Phillips W, Gault MH (1990). Charcot-Marie-tooth disease and nephropathy in a mother and daughter with a review of the literature. Nephron..

[13] De Rechter S, De Waele L, Levtchenko E, Mekahli D (2015). Charcot-Marie-tooth: are you testing for proteinuria. Eur J Paediatr Neurol.

[14] Rodriguez PQ, Lohkamp B, Celsi G, Mache CJ, Auer-Grumbach M, Wernerson A (2013). Novel INF2 mutation p. L77P in a family with glomerulopathy and Charcot-Marie-tooth neuropathy. Pediatr Nephrol.

[15] Chhabra ES, Ramabhadran V, Gerber SA, Higgs HN (2009). INF2 is an endoplasmic reticulum-associated formin protein. J Cell Sci.

[16] Lee HK, Han KH, Jung YH, Kang HG, Moon KC, Ha IS (2011). Variable renal phenotype in a family with an INF2 mutation. Pediatr Nephrol.

[17] Barua M, Brown EJ, Charoonratana VT, Genovese G, Sun H, Pollak MR (2013). Mutations in the INF2 gene account for a significant proportion of familial but not sporadic focal and segmental glomerulosclerosis. Kidney Int.

